# Role of Carbonic Anhydrase IV in the Bicarbonate-Mediated Activation of Murine and Human Sperm

**DOI:** 10.1371/journal.pone.0015061

**Published:** 2010-11-24

**Authors:** Petra M. Wandernoth, Michael Raubuch, Nadja Mannowetz, Holger M. Becker, Joachim W. Deitmer, William S. Sly, Gunther Wennemuth

**Affiliations:** 1 Department of Anatomy and Cell Biology, Saarland University, Homburg, Saar, Germany; 2 Division of Zoology/Membrane Transport, Department of Biology, University of Kaiserslautern, Kaiserslautern, Germany; 3 Division of General Zoology, Department of Biology, University of Kaiserslautern, Kaiserslautern, Germany; 4 Edward A. Doisy Department of Biochemistry and Molecular Biology, Saint Louis University School of Medicine, St. Louis, Missouri, United States of America; Universidade Federal do Rio de Janeiro, Brazil

## Abstract

HCO_3_^−^ is the signal for early activation of sperm motility. *In vivo*, this occurs when sperm come into contact with the HCO_3_^−^ containing fluids in the reproductive tract. The activated motility enables sperm to travel the long distance to the ovum. In spermatozoa HCO_3_^−^ stimulates the atypical sperm adenylyl cyclase (sAC) to promote the cAMP-mediated pathway that increases flagellar beat frequency. Stimulation of sAC may occur when HCO_3_^−^ enters spermatozoa either directly by anion transport or indirectly via diffusion of CO_2_ with subsequent hydration by intracellular carbonic anhydrase (CA). We here show that murine sperm possess extracellular CA IV that is transferred to the sperm surface as the sperm pass through the epididymis. Comparison of CA IV expression by qRT PCR analysis confirms that the transfer takes place in the corpus epididymidis. We demonstrate murine and human sperm respond to CO_2_ with an increase in beat frequency, an effect that can be inhibited by ethoxyzolamide. Comparing CA activity in sperm from wild-type and CA IV^−/−^ mice we found a 32.13% reduction in total CA activity in the latter. The CA IV^−/−^ sperm also have a reduced response to CO_2_. While the beat frequency of wild-type sperm increases from 2.86±0.12 Hz to 6.87±0.34 Hz after CO_2_ application, beat frequency of CA IV^−/−^ sperm only increases from 3.06±0.20 Hz to 5.29±0.47 Hz. We show, for the first time, a physiological role of CA IV that supplies sperm with HCO_3_^−^, which is necessary for stimulation of sAC and hence early activation of spermatozoa.

## Introduction

Post-testicular maturation of sperm in the epidydimis and the female genital tract includes multiple changes in sperm membrane composition and signal transduction [1], [2], [3], [4]. After spermatogenesis sperm travel along the epididymal tract and the female genital tract where they undergo fundamental changes in their motility. During the early passage they have to adjust from immotility to a linear swimming behaviour to travel along the vagina, uterus and the oviduct. Bicarbonate plays a major role in this early activation of spermatozoa and is necessary for successful fertilization [5], [6], [7]. The activation of soluble adenylyl cyclase (sAC) by bicarbonate induces the elevation of intracellular cAMP and subsequent phosphorylation of several proteins by protein kinase A. We have previously shown that bicarbonate in concentrations of 15 mM induces a reversible, robust acceleration of sperm resting beat frequency from 2–3 Hz to 7 Hz in less than 1 min. In addition, acceleration of beat frequency is accompanied by facilitation of Ca^2+^ channels and beat symmetry [8].

Little is known how sperm regulate a rise of intracellular bicarbonate. In addition to bicarbonate transporters using HCO_3_^−^ of the oviduct as a substrate, carbonic anhydrases (CAs) can catalyze the equilibrium between CO_2_ and HCO_3_^−^. More than a dozen CAs have been identified in mammals [9]. In this study we focused on the physiological role of CA IV after it was identified in the male reproductive tract in mouse and rat [10], [11], [12] and linked it to the early activation of sperm motility by bicarbonate. We show that extracellular carbonic anhydrase IV is a key enzyme in the early activation of sperm. In addition, we demonstrate that sperm do not acquire CA IV during spermatogenesis, but instead CA IV is transferred to the sperm membrane during the passage through the corpus epididymidis.

## Materials and Methods

### Ethics statement

Killing of animals was applied for and approved by the animal rights office of the Saarland University (ID 18/08). Human sperm were collected from healthy volunteers with approval of the local ethics committee of the Philipps-University of Marburg, Germany (approval Number 105/05). Written and informed consent was obtained from all participants.

### Animals and Cell Preparation

Sperm were isolated from NMRI mice, C57BL/6J mice and CA IV^−/−^ (B6.129S1-Car^4tm1Sly/J^). After treatment with isoflurane and cervical dislocation, the caput, corpus, cauda epididymidis and vasa deferentia were excised from mice, transferred into 1 ml HS buffer, incised several times, and incubated for 20 min at 37°C in 5% CO_2_. Released sperm were washed twice (400× g for 3 min) and stored at 1−2×10^7^ cells/ml in HCO_3_^−^-free HS buffer (in mM): 135 NaCl, 5 KCl, 2 CaCl_2_, 1 MgCl_2_, 20 HEPES, 5 glucose, 10 lactic acid, 1 pyruvic acid, adjusted to pH 7.4 with NaOH. Each experiment was performed with three or more animals.

#### Preparation of human sperm

Human ejaculates were obtained from healthy volunteers between 20 and 25 years of age. The fresh ejaculates were centrifuged (300× g, 3 min) and the pellet was split into two samples. Each pellet was transferred to 40 ml buffer HS and incubated for 2 hours at room temperature to remove HCO_3_^−^ from the seminal plasma. After centrifugation, the cells were pooled and re-suspended in 0.5 ml buffer HS and stored at 3−4×10^7^ cells/ml.

### Immunohistochemistry

Sperm from caput, corpus and cauda epididymidis were prepared as described above. After washing in HS buffer, sperm suspensions were diluted to a final concentration of 4×10^5^ cells/ml. Sperm were air-dried on cover slips, fixed for 15 min in methanol and again air-dried.

Mouse kidney, testis, epididymis and vas deferens were fixed for 6 h in Bouin solution, dehydrated and embedded in paraffin. For immunoreactions, tissue was cut into 5 µm slices and dried on glass slides prior to deparaffinization with xylol and rehydration in a descending alcohol series (100, 90, 80, and 70%). Endogenous peroxidase activity was blocked for 45 min at 37°C by treatment with glucose oxidase (Sigma, Steinheim, Germany) in PBS-glucose buffer (10 mM glucose, 1 mM NaN_3_ and 0.4 U/ml glucose oxidase). The slices were incubated overnight at 4°C with goat anti-CA IV IgG (R&D Systems, Minneapolis, MN, USA), diluted 1∶100 in PBS/5% BSA/avidin (1∶300) (Merck, Darmstadt, Germany), washed twice for 5 min in PBS and incubated for 30 min at room temperature with the secondary biotinylated rabbit anti goat-IgG (Vector Laboratories, Burlingame CA, USA) diluted 1∶200 in PBS/5% BSA/biotin (1∶50) (Sigma, Steinheim, Germany). Finally, the slides were washed twice for 5 min in PBS. For signal enhancement the Vectastain® kit (Linaris, Wertheim-Bettingen, Germany) was applied for 30 min at room temperature according to the manufacturer's protocol. Histochemical localization of CA IV immunoreactivity was performed using diaminobenzidine (DAB) (Sigma, Steinheim, Germany) as a chromogen. The evaluation of the specificity of the immunoreactivity is based on a comparison of tissues from wild-type and CA IV^−/−^ mice. For nuclear staining, the slides were treated for 1 min with hematoxylin (Roth, Karlsruhe, Germany), followed by 5 min incubation in tap water to induce the color reaction. Finally, the slides were dehydrated and mounted with DEPEX (Serva, Heidelberg, Germany). Analysis was performed with a light microscope (Axiophot, Zeiss, Jena, Germany).

### Western Blot

Mouse kidney, testis, epididymis and vas deferens were isolated and minced in homogenization buffer (100 mM NaCl, 10 mM HEPES, 2 mM EDTA, 1 mM DTT, 2% Triton X-100) on ice. Samples were kept on ice for 30 min and protein fractions were extracted by centrifugation several times for 15 min, at 11,000× g and 4°C. The protein concentrations were determined photometrically with a BCA Protein Assay Kit (Thermo Scientific, Rockford, IL, USA). Protein samples were diluted 1∶1 with 2X-Laemmli buffer and stored at −20°C.

Protein of suspensions of sperm from all three regions of the epididymis and vas deferens were extracted by the addition of an equal volume of 2X-Laemmli buffer. The extracts were clarified by centrifugation at 13,000 rpm for 15 min at 4°C.

For western blot analysis, the extracts from 100 µg (tissue) or 30 µl (sperm suspension) were adjusted to 5% mercaptoethanol. The samples were boiled for 5 min (100°C) and separated by SDS-Page. After immunoblotting and blocking with TBS/5% Slim-Fast™ (Allpharm, Messel, Germany), the membrane was incubated overnight at 4°C with goat anti-CA IV IgG (1∶1,000 in TBS-T) (R&D Systems, Minneapolis, MN, USA). After washing thrice with TBS-T, the membranes were incubated with HRP-conjugated donkey anti-goat IgG (diluted 1∶10 000 in TBS-T) for 1 h at RT. Proteins were detected with an ELC detection reagent (GE Healthcare, Buckinghamshire, UK) on a Chemi-Doc™ XRS+ apparatus (Bio Rad, München, Germany).

### qRT PCR

Tissue isolated from kidney, testis, caput, corpus and cauda epididymidis was homogenized in 50 µl Tri-Fast™ (PeqLab, Erlangen, Germany) on ice. Total RNA was extracted with the RNeasy Plus™ Micro Kit (50) (Quiagen, Hilden, Germany) and cDNA was prepared with the High Capacity cDNA™ Reverse Transcription Kit (Applied Biosystems, Foster City, CA, USA). To detect the CA IV gene, 100 ng of total cDNA were processed with a TaqMan^®^ gene expression assay (Applied Biosystems, Foster City, CA, USA). For relative quantitation with the ΔΔC_t_ method [13], we used 18S ribosomal RNA as endogenous control and kidney as reference tissue. All measurements were carried out on a StepOnePlus™ qRT-PCR device from Applied Biosystems (Foster City, CA, USA). Results are presented as mean RQ values ± SEM from three independent preparations.

### Assessment of Viability and Motility Parameters

Sperm motility parameters were assessed by means of a computer-assisted sperm analysis (CASA) system (MedeaLAB CASA System, v 5.5, Medical Technology GmbH, Altdorf, Germany). The parameters measured were average velocity [µm/s], motility [%] and the proportions of fast and slow progressive sperm [%].

After washing, sperm were stored in pre-warmed HS buffer containing 5% BSA. For analysis, 20 µl of the sperm suspension was loaded into a pre-warmed (37°C) counting chamber (Makler, Sefi-Medical Instruments ltd., Biosigma S.r.I., Italy). The results are presented as mean ± SEM.

### Waveform Analysis

The flagellar waveform was analyzed as previously described [14] with a Nikon Diaphot 300 microscope. In brief, images were collected at 150 Hz (murine sperm) and 300 Hz (human sperm) respectively by a M3 high speed camera (IDT; Tallahassee, FL, USA). Determination of flagellar beat frequency was performed by semi-automated analysis software written in Igor-Pro™ (Wavemetrics, Lake Oswego OR, USA). The data obtained was collected in Sigma Plot (Systat Software, San Jose, CA, USA) and presented as mean ± SEM. Sp-5,6-dichloro-1-β-D-ribofuranosylbenzimidazole-3′,5′-monophosphorothioate (cBIMPS) was supplied by BioMol (Hamburg, Germany), ethoxyzolamide (EZA) and acetazolamide (AZA) were from Sigma-Aldrich (Steinheim, Germany). 2% and 5% CO_2_ was supplied by Air Liquide (Düsseldorf, Germany). To maintain CO_2_ equilibration during measurements a heated measuring chamber was continuously perfused with CO_2_. Where indicated CO_2_ was additionally applied to solutions by gas bubbler manifolds (Harvard Aparatus, Kent, UK) to allow equilibration before perfusion.

### Determination of CA Activity by Mass Spectrometry

Determination of CA activity was performed as previously described [15]. In brief, we monitored ^18^O depletion from doubly-labeled ^13^C^18^O_2_ through several hydration and dehydration steps of CO_2_ and HCO_3_^−^ at 25°C [16], [17]. The loss of ^18^O from ^13^C^18^O^18^O (m/z = 49) over the intermediate product ^13^C^18^O^16^O (m/z = 47) and the end product ^13^C^16^O^16^O (m/z = 45) was observed with a quadrupole mass spectrometer (MSD 5970; Hewlett Packard, Waldbronn, Germany). The relative ^18^O enrichment was documented by the constant measurement of the changes in the signals for m/z = 45, m/z = 47, m/z = 49 (a_45_, a_47_, a_49_) over time and was calculated by the following equation: log enrichment  =  log (a_49_×100/(a_49_+ a_47_+a_45_)). The linear slope of the log enrichment over time, calculated with OriginPro™ 7 (OriginLab, Northamton, MA), provided the rate of loss of ^18^O. This was used to calculate the carbonic anhydrase activity, by comparing the rate with the corresponding rate of the non-catalyzed reaction. To calculate the enzyme activity in units, the Badger and Price [18] definition was used, which defines 1 unit of activity as producing a 100% increase in the non-catalyzed rate of ^18^O depletion from doubly-labeled ^13^C^18^O_2_. For the experiments, a cuvette was filled with 8 ml HS buffer, followed by 100–200 µl sperm suspension (4×10^6^ cells). EZA was added after 6 minutes in the respective concentration.

### Statistics

Student's *t*-Test was used to calculate the significance in differences of mean values. In the figures shown a significant level of p<0.05 is marked with *, p<0.01 is marked with **, and p<0.001 with ***.

## Results

**Figure 1:**
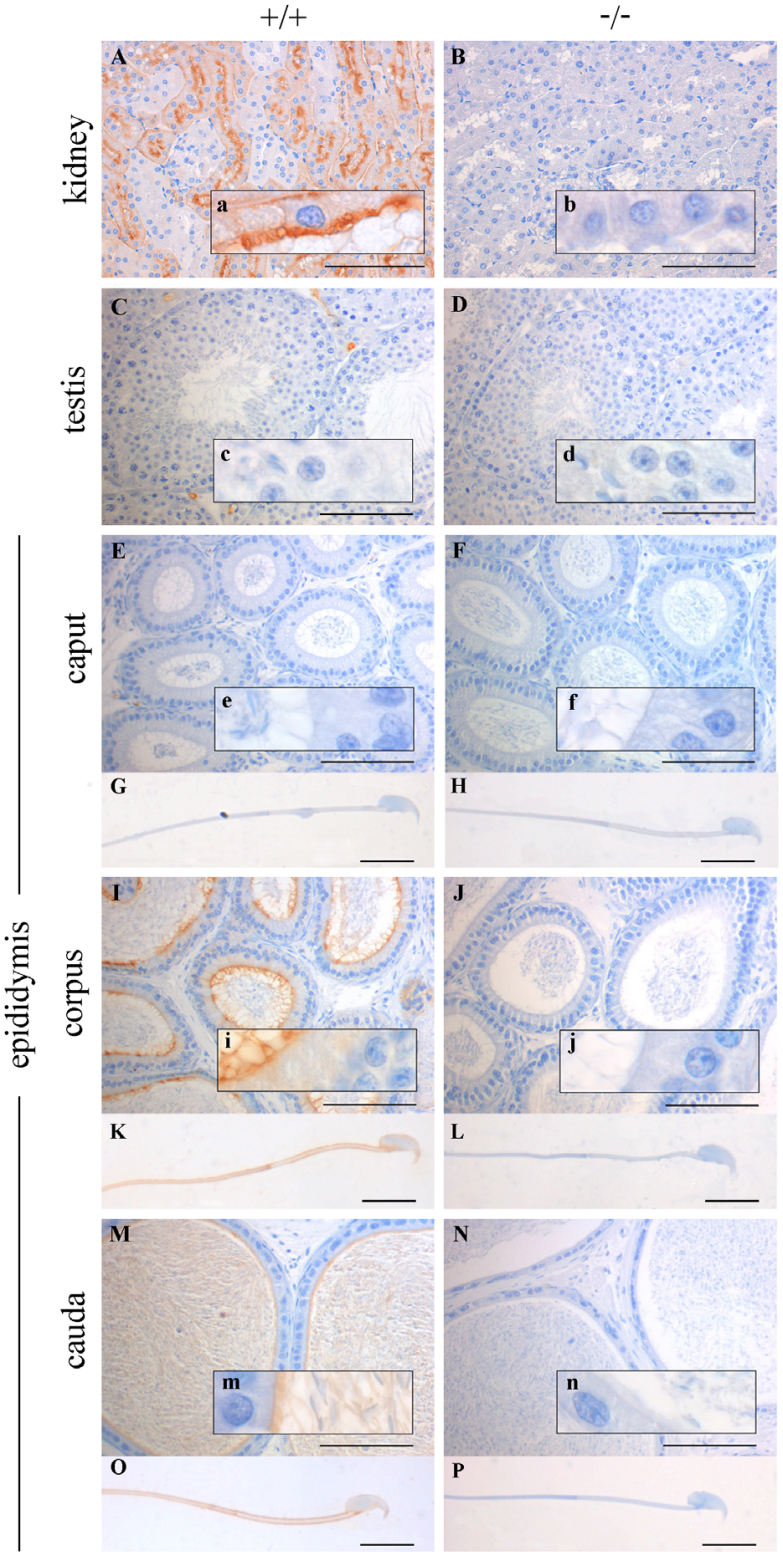
Immunohistochemical localization of CA IV. Immunohistochemical localization of CA IV in wild-type (+/+) and CA IV^−/−^ (−/−) tissue slices and isolated sperm from different epididymis sections. Wild-type tissue from kidney, corpus and cauda epididymidis (**A**, **I, M**) show immunoreactions. Kidney shows staining in the apical and basal plasma membrane of proximal tubuli. Corpus and cauda epididymidis display the signal in the stereocilia network. In addition, sperm of the cauda are also CA IV positive. No signal is present in the wild-type testis and caput epididymidis (**C, E**) or in any of the −/− tissues (**B**, **D**, **F**, **J**, **N**) Wild-type corpus and cauda sperm (**K, O**) show immunostaining in the plasma membrane along the tail and the head. No signal is detectable in the wild-type caput sperm (**G**) or in any of the CA IV^−/−^ sperm (**J, L, P**). (bar: tissue = 100 µm; sperm = 10 µm).

Wild-type kidney (the positive control) shows immunoreactions with the CA IV antibody in the apical plasma membrane of the proximal tubules in the cortex of the kidney (Fig. 1A/a). A weaker signal is detectable on the basal plasma membrane of the tubulus. CA IV staining is not found in either wild-type testis or caput epididymidis (Fig. 1C/c; 1E/e). However, immunoreactivity is visible in the apical plasma membrane of epithelial cells in the corpus epididymidis (Fig. 1I/i). At higher magnification (insets) the CA IV signal is located in the stereocilia network. In addition we found immunoreactions with CA IV antibodies in both the stereocilia network and spermatozoa of cauda epididymids (Fig. 1M/m). The CA IV^−/−^ tissue is completely negative for CA IV (Fig. 1B/b; 1D/d; 1F/f; 1J/j; 1N/n).

Both wild-type tissue and caput sperm show no immunoreactions with CA IV antibodies (Fig. 1G), whereas specific staining is found in corpus (Fig. 1K) and cauda sperm (Fig. 1O) in the plasma membrane along the whole sperm tail and near the plasma membrane of the head. No signal is found in CA IV^−/−^ sperm (Fig. 1H/L/P).

**Figure 2:**
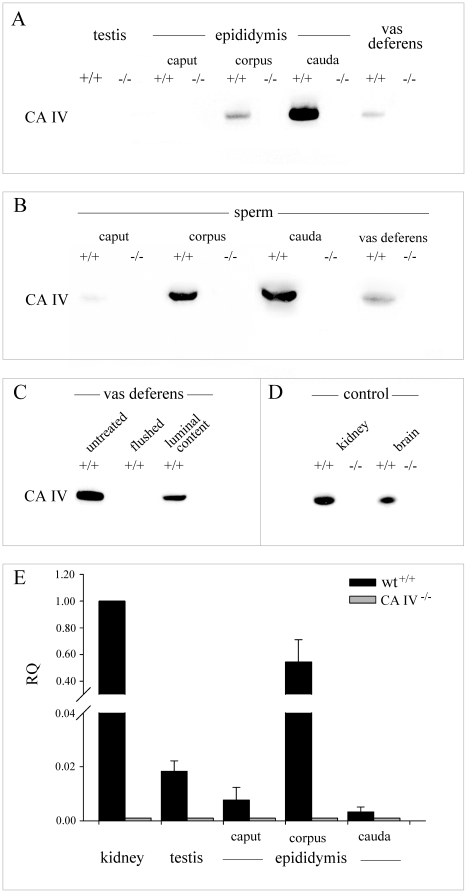
Immunoblot and real-time PCR of CA IV. **A**, Immunoblot of CA IV. A CA IV signal in the range of 38 kDa is present in wild-type corpus and cauda epididymidis and vas deferens. No specific CA IV band is detectable in wild-type testis and caput epididymidis or in any of the CA IV^−/−^ tissues. **B**, Analysis of sperm protein fractions isolated from the different sections of the epididymidis shows a positive signal in corpus and cauda sperm and sperm from vas deferens. No specific signal is present in wild-type caput sperm or in any of the CA IV^−/−^ sperm. **C**, CA IV is present in the whole vas deferens tissue and not present in the flushed vas deferens. With the luminal content only a specific CA IV band can be seen. **D**, kidney and brain tissue were used as positive control. **E**, CA IV qRT PCR analysis of wild-type and CA IV^−/−^ mice. The diagram shows mean RQ values ± s.e.m. of three independent experiments for each tissue. In relation to wild-type kidney (calibrator) the RQ value of wild-type corpus epididymidis averages at 0.54. No CA IV mRNA is detectable in the other wild-type or in any of the CA IV^−/−^ tissues (n = 3).of wild-type and CA IV^−/−^ mice.

Figure 2A shows immunoblots for protein extracts of wild type caput, corpus, and cauda epididymidis and the vas deferens. A single ∼38 kDa immunoreactive CA IV band is detectable in corpus, cauda epididymidis and the vas deferens but is absent in caput epididymidis and whole testis. No signal is detected in tissue from CA IV^−/−^ mice. For wild type mice, extracts of corpus and cauda sperm and sperm from vas deferens show a prominent 38 kDa immunoreactive CA IV band (Fig. 2B). A small signal is detectable in caput sperm. CA IV^−/−^ sperm do not show any CA IV signal. Tissue of flushed vas deferens and sperm were examined separately and the results demonstrate, that CA IV is localized in luminal sperm only (Fig. 2C). Kidney and brain were used as positive controls (Fig. 2D).

qRT PCR analysis was used to examine the expression of CA IV mRNA in the male reproductive tract (Fig. 2E). CA IV^+/+^ and CA IV^−/−^ tissue from kidney, testis, caput, corpus and cauda epididymidis was analyzed. Kidney, as the reference tissue, was assigned a constant RQ value of 1. Wild-type corpus epididymidis shows a significant RQ signal of 0.54. No significant RQ signal was found in the other wild type tissues or in any of the CA IV^−/−^ tissues.

**Figure 3:**
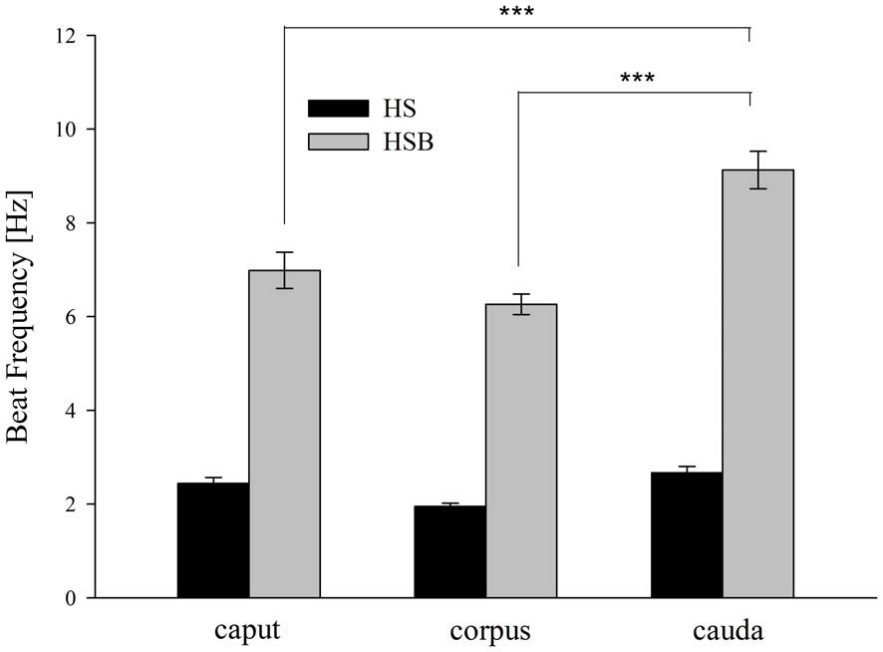
Response of sperm to bicarbonate from different epididymal segments. Sperm of different segments of epididymis show varying responses to bicarbonate. Values shown are mean ± S.E.M. Sperm in HS buffer (black bars) do not show significant differences in resting beat frequency. Mean values were 2.42±0.13 Hz for caput sperm, 1.95±0.07 Hz for corpus sperm and 2.67±0.13 Hz for sperm of cauda epididymidis. Sperm beat frequency in HSB buffer (containing 15 mM HCO_3_^−^) (gray bars) increases the beat frequencies to 6.79±0.42 Hz for caput sperm, 6.09±0.25 Hz for corpus sperm and 9.13±0.40 Hz for sperm of cauda epididymidis. (*n* = 30).

Figure 3 compares the ability of bicarbonate to increase the flagellar beat frequency of sperm from the caput, corpus and cauda epididymidis. Stimulation with 15 mM bicarbonate leads to an increase in beat frequency of randomly-selected cells from 2.42±0.13 Hz to 6.79±0.42 Hz for caput sperm and from 1.95±0.07 Hz to 6.09±0.25 Hz for corpus sperm, whereas for cauda sperm the beat frequency increases from 2.67±0.13 Hz to 9.13±0.40 Hz.

**Figure 4:**
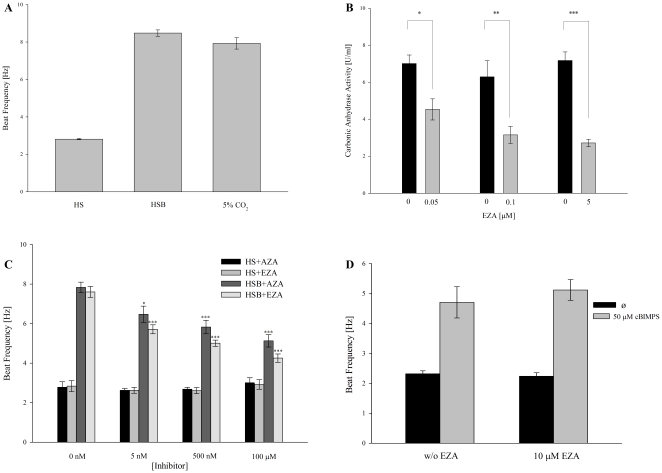
Effect of CA-Inhibitors on sperm beat frequency and CA-activity. **A**, The effect of HCO_3_^−^ und CO_2_ on sperm beat frequency. Values shown are mean ± S.E.M. Mouse sperm beat frequency was measured in HS buffer, HSB buffer (containing 15 mM HCO_3_^−^) and in HS buffer in the presence of 5% CO_2_. Mean values are 2.96±0.17 Hz of sperm in HS buffer, 8.48±0.17 Hz of sperm in HSB buffer and 7.94±0.31 Hz for sperm stimulated with CO_2_. (*n* = 10). **B**, Concentration-dependent inhibition of carbonic anhydrase activity was determined by mass spectrometry. The addition of varying EZA concentrations results in a decrease of enzymatic activity of between 35.0% (4.53±0.87 U/ml) for 50 nM EZA and 62.12% (2.72±0.12 U/ml) for 5 µM EZA (*n* = 6). **C**, Sperm beat frequency was measured in HS and HSB buffer in the absence or presence of different EZA or AZA concentrations. Resting beat frequency in HS is not influenced by EZA or AZA. In the presence of bicarbonate, the addition of 100 µM EZA decreases sperm beat frequency from 7.60±0.28 Hz to 4.26±0.21 Hz, whereas the addition of 100 µM AZA decreases beat frequency from 7.84±0.27 Hz to 5.13±0.31 Hz (*n* = 10). **D**, The cAMP analogon cBIMPS increases sperm beat frequency by acting downstream of carbonic anhydrases. Sperm measured in HS buffer including cBIMPS in the absence or presence of 10 µM ethoxyzolamide (EZA). (*n* = 10).

Figure 4A shows that both bicarbonate and CO_2_ increase flagellar beat of cauda sperm similarly. Sperm accelerate their beat frequency from 2.96±0.17 to 8.48±0.17 Hz when treated for 5 min with HS medium containing 15 mM bicarbonate. The application of 5% CO_2_ to sperm in HS medium alone increases the beat frequency within 5 minutes to 7.94±0.31 Hz. The total carbonic anhydrase activity of ∼4×10^6^ cells was determined by mass spectrometry before and after the application of different concentrations of the carbonic anhydrase inhibitor EZA (Fig. 4B). The addition of 50 nM EZA significantly decreases enzymatic activity from 7.01±0.46 U/ml to 4.53±0.87 U/ml (35.38% reduction), 100 nM EZA leads to a highly significant decrease from 6.29±0.57 U/ml to 3.15±0.46 U/ml (49.92% reduction) and 5 µM EZA reduces enzymatic activity from 7.18±0.20 U/ml to 2.72±0.12 U/ml (62.12% reduction). EZA or AZA also produced a dose-dependent reduction in the action of HCO_3_^−^ on the flagellar beat. Figure 4C shows that after treatment with 5 nM or 500 nM EZA, bicarbonate increases the beat frequency from 2.63±0.15 Hz to 5.71±0.23 Hz and from 2.61±0.15 Hz to 5.01±0.16 Hz. The addition of HCO_3_^−^ in the presence of 100 µM EZA results in a <1.5-fold increase in sperm beat frequency (from 2.92±0.24 Hz to 4.26±0.21 Hz) only. Treatment with AZA in the same concentrations as EZA shows nearly the same inhibitory effect (5 nM AZA: from 2.63±0.10 Hz to 6.47±0.41 Hz; 500 nM AZA: from 2.68±0.10 Hz to 5.83±0.34 Hz; 100 µM AZA: from 3.01±0.25 Hz to 5.13±0.31 Hz). To examine if EZA has actions downstream of the action of HCO_3_^−^, we used cBIMPS to stimulate sperm both in the absence and in the presence of EZA. In the absence of EZA, sperm beat frequency is increased within 10 minutes from 2.32±0.10 Hz to 4.71±0.52 Hz after application of 50 µM cBIMPS (Fig. 4D). Sperm which were treated with 10 µM EZA increase their beat frequency to a similar extent (from 2.24±0.11 Hz to 5.12±0.35 Hz).

**Figure 5:**
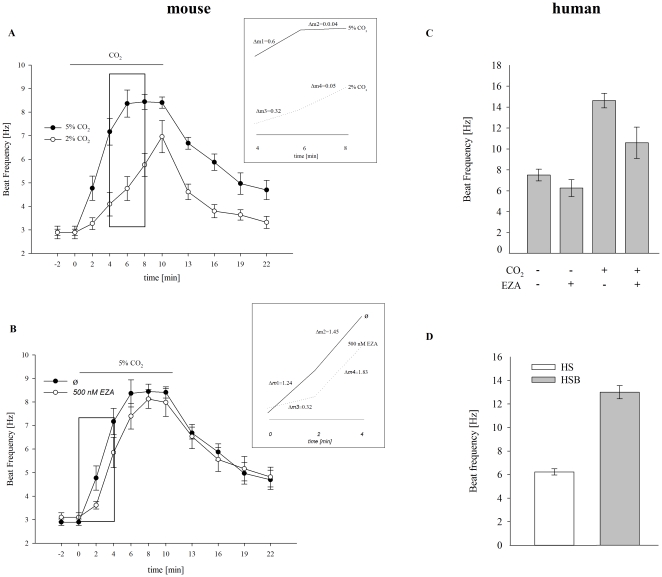
Effect of EZA and bicarbonate on human sperm beat frequency. **A**, The acceleration of mouse sperm beat frequency varies according to the CO_2_ concentration. Sperm beat frequency was measured in HS buffer, which corresponds to atmospheric CO_2_ concentration, followed by 2% CO_2_ (gray line) and 5% CO_2_ (black line) application for 10 minutes. 6 minutes after the application of 5% CO_2_, the beat frequency is increased from 2.90±0.14 Hz to 8.37±0.58 Hz, while it takes 10 minutes for the frequency to increase from 2.89±0.27 Hz to 6.96±0.69 Hz by the application of 2% CO_2_ (*n* = 10). The beat frequencies from the dashed box are shown on an expanded time scale in A and B. **B**, EZA inhibits the accelerating effect of CO_2_ on mouse sperm beat frequency. Sperm beat frequency was measured in HS buffer (black line) and HS buffer containing 500 nM EZA (gray line), followed by 5% CO_2_ application for 10 minutes. 6 minutes after CO_2_ application in the absence of EZA, the beat frequency increases from 2.90±0.14 Hz to 8.37±0.58 Hz. In the presence of EZA beat frequency reaches a maximal value of 8.13±0.40 Hz after 8 minutes of CO_2_ application (*n* = 10). **C**, Human sperm respond towards CO_2_ in an EZA sensitive way. Sperm were stimulated for 40 s with 2% CO_2_ either in the absence or presence of 1 µM EZA. Without the inhibitor, beat frequency rises from 7.51±0.56 Hz to 14.62±0.70 Hz. With EZA, sperm speed from 6.26±0.81 Hz to 10.59±1.50 Hz. (*n* = 10). **D**, Human sperm respond to HCO_3_^−^. Sperm were stimulated for 60 s with 15 mM HCO_3_^−^. During that time, the resting beat frequency increases from 6.23±0.26 Hz to 13.00±0.56 Hz. (*n* = 13). In all panels, results are presented as mean values±SEM.

Different concentrations of CO_2_ increase the flagellar beat time-dependently (Fig. 5A). The stimulation of sperm with 5% CO_2_ accelerates their beat frequency within 6 minutes from 2.90±0.14 Hz to 8.37±0.58 Hz. The slope of the beat frequency between the 4^th^ and the 6^th^ minute after application of 5% CO_2_ was 0.60 Hz/min (Δm_1_), whereas between the 6^th^ and 8^th^ minute a slope of 0.04 Hz/min (Δm_2_) was determined (dashed box). The application of 2% CO_2_ increased beat frequency within 6 minutes from 2.89±0.27 Hz to 4.76±0.51 Hz only. For the time period between the 4^th^ and the 6^th^ minute after the application of 2% CO_2_ a slope of 0.33 Hz/min (Δm_3_) was determined, which was increased in the following two-minute period to 0.50 Hz/min (Δm_4_).

In the absence of EZA, 5% CO_2_ accelerates sperm beat frequency within 6 minutes from 2.90±0.14 Hz to 8.37±0.58Hz (Fig. 5B). In the first two minutes after CO_2_ application, beat frequency rises with a slope of 1.24 Hz/min, which is increased to 1.45 (Δm_2_) within the next two-minute period. In the presence of 0.5 µM EZA, beat frequency accelerates from 3.10±0.10 Hz to 7.40±0.54 Hz within 6 minutes. Within the first two minutes of CO_2_ application beat frequency increases with a slope of 0.32 Hz/min (Δm_3_) in the presence of EZA (dashed box). In the following two-minute period the slope of beat frequency is increased to 1.83 Hz/min (Δm_4_) (n = 10).

Flagellar movement of human sperm was analyzed in the same way as for murine sperm. With 2% CO_2_, the resting beat frequency of 7.51±0.56 Hz speeds to 14.62±0.70 Hz (Fig. 5C) after 40 s, whereas in the presence of 1 µM EZA, beat frequency rises from 6.26±0.81 Hz to 10.59±1.50 Hz. Also with 15 mM bicarbonate, which was applied for 60 s, an accelerated beat frequency from 6.23±0.26 Hz to 13.00±0.56 Hz is apparent (Fig. 5D).

**Figure 6:**
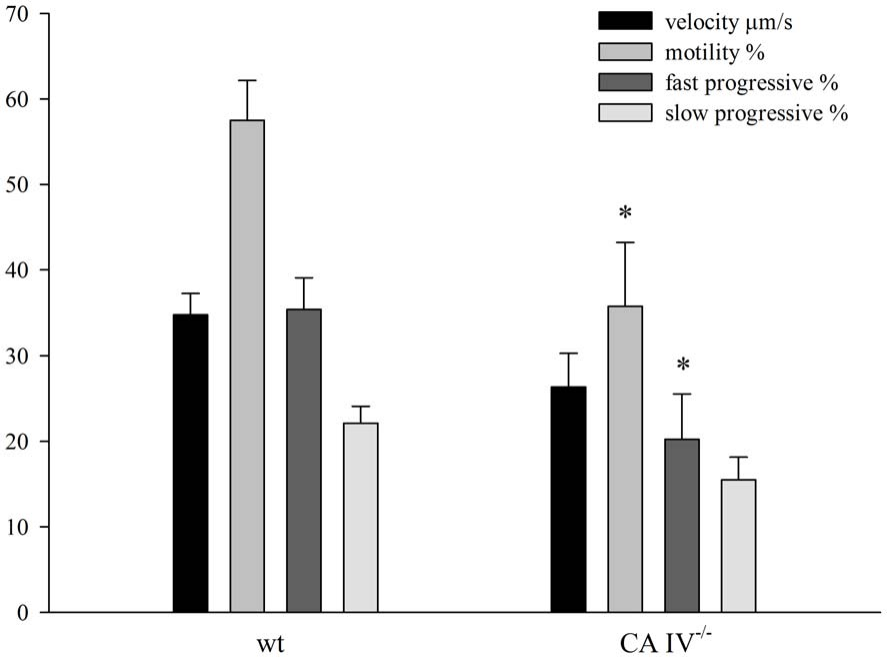
CASA of sperm from CA ^−/−^ mice. Differences in motility parameters between wild-type sperm and sperm of CA IV^−/−^ mice. Sperm motility parameters were determined by using a computer-assisted sperm analysis (CASA) system. In comparison to wild-type sperm the number of motile and the quantity of fast progressive sperm of CA IV^−/−^ is significantly reduced (wild-type: 57.50±4.67%; 35.38±3.69%; CA IV^−/−^: 35.75±7.46%; 20.25±5.29%) No difference between sperm of wild-type and CA IV^−/−^ is detectable in average velocity and slow progressive motility (n = 8).

Figure 6 compares the motility parameters of CASA determined for sperm of wild type and of CA IV^−/−^ mice in the absence of bicarbonate. In CA IV^−/−^, the total motility is significantly decreased (35.75±7.46%) as compared to wild-type sperm (57.50±4.67%). In addition, the amount of fast progressive sperm is also significantly lower in CA IV^−/−^ mice (20.25±5.29% as compared to 35.38±3.69%). No significant changes is observed in the comparison of the average velocity of CA IV^−/−^ and wild-type sperm (wild-type: 34.75±2.49 µm/s; CA IV^−/−^: 26.38±3.93 µm/s). There are also no significant changes in slow progressive sperm from wild-type mice and CA IV^−/−^ mice (wild-type: 22.13±1.97%; CA IV^−/−^: 15.50±2.65%).

**Figure 7:**
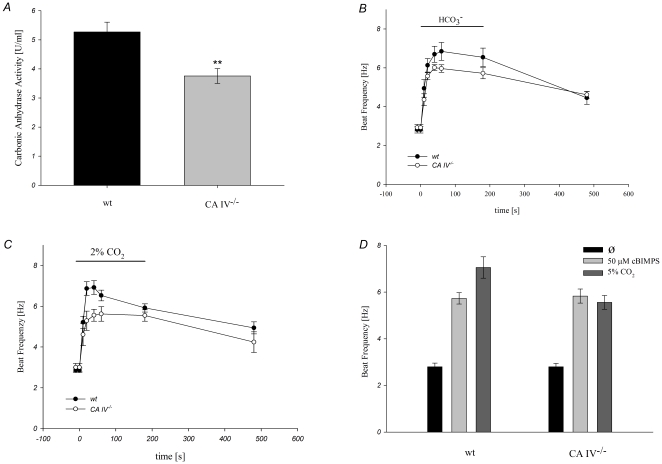
Sperm beat frequency and CA-activity in CA ^−/−^ mice. **A**, Enzymatic activity of CA measured by mass spectrometry. Mean value of enzymatic activity of wild type sperm, is 5.26±0.34 U/ml, whereas sperm of CA IV^−/−^ mice show activity of 3.57±0.25 U/ml only (*n* = 6). **B**, Sperm of CA IV^−/−^ mice show a decreased response to HCO_3_^−^. Within the first 20 seconds after the addition of bicarbonate, the beat frequency of wild-type sperm (solid line) increases from 2.81±0.17 Hz to 6.13±0.35 Hz and frequency of CA IV^−/−^ sperm from 2.92±0.17 Hz to 5.57±0.18 Hz (*n* = 10). **C**, Sperm of CA IV^−/−^ mice show a decreased response to CO_2_. Through the application of 2% CO_2_ in the measuring chamber and the manifolds wild-type sperm beat frequency (solid line) increase within the first 20 seconds from 2.86±0.12 Hz to 6.87±0.34 Hz, while the beat frequency of CA IV^−/−^ sperm takes 1 min to rise from 3.06±0.20 Hz to 5.29±0.47 Hz. (*n* = 10). **D**, Sperm of CA IV^−/−^ mice show the same response to the cAMP analogon cBIMPS as wild-type sperm. 20 seconds after stimulation with 5% CO_2_, the sperm of CA IV^−/−^ mice show a 20.48% reduction in beat frequency compared to wild-type sperm. By contrast, after 10 minutes stimulation with 50 µM cBIMPS wild-type and CA IV^−/−^ sperm speed their beat to comparable values (from 2.89±0.16 Hz to 5.71±0.24 Hz and from 2.80±0.15 Hz to 5.83±0.30 Hz, respectively)(*n* = 10).

In comparison to wild type sperm, the enzymatic activity of CA IV^−/−^ animals is decreased highly significantly by 32.13% (from 5.26±0.34 U/ml to 3.57±0.25 U/ml) (Fig. 7A). Waveform analysis was performed to analyze the effect of HCO_3_^−^ and CO_2_ on the beat frequency of CA IV^−/−^ sperm (Fig. 7B, C). Fig. 7B shows that there is only a slight difference in the first 20 seconds between the sperm of wild-type and CA IV^−/−^ animals in the response to HCO_3_^−^. Perfusion of sperm of wild-type and CA IV^−/−^ animals with buffer containing 15 mM HCO_3_^−^ leads to an increase of sperm beat frequency within 20 seconds from 2.81±0.17 Hz to 6.13±0.35 Hz and from 2.92±0.17 Hz to 5.57±0.18 Hz, respectively (n = 10).

Sperm of wild-type animals increase their beat frequency from 2.86±0.12 Hz to 6.87±0.34 Hz within 20 seconds after 2% CO_2_ application, whereas CA IV^−/−^ sperm accelerate their beat from 3.06±0.20 Hz to 5.29±0.47 Hz (Fig. 7C). Compared to wild-type sperm, sperm of CA IV^−/−^ animals show a significant reduction of beat frequency by 20.48% after stimulation with 5% CO_2_ for 20 seconds (wild-type: 7.08±0.43 Hz: CA IV^−/−^; 5.63±0.36 Hz). By contrast, after 10 minutes of stimulation with 50 µM cBIMPS, wild-type and CA IV^−/−^ sperm speed their beat to values, which do not show significant differences (from 2.89±0.16 Hz to 5.71±0.24 Hz and from 2.80±0.15 Hz to 5.83±0.30 Hz, respectively) (Fig. 7D).

## Discussion

This work analyses the distribution and physiological activity of CA IV in the murine male genital tract. The results show that CA IV is involved in the regulation of intracellular bicarbonate concentration and early activation of spermatozoa by bicarbonate. Bicarbonate as an important factor for sperm maturation and storage in the male reproductive tract, is responsible for acceleration of sperm beat frequency and calcium channel activation [3], [8], [19].

### CA IV has a distinct location in the male reproductive tract

By immunohistochemistry we show that CA IV is not a constituent that is acquired during spermatogenesis. CA IV was not detectable at all stages of sperm maturation in testis. However, in caput, corpus and cauda epididymidis, CA IV appears in the stereocilia of epithelium and from there on also in isolated spermatozoa. Localization in the plasma membrane and in the stereocilia network are in accordance with the findings that CA IV is an extracellular GPI-anchored protein [20]. This supports the proposal of Ekstedt and co-workers [12] who recognized the possible transfer of CA IV during sperm passage through the epididymal tract. In rat, a different distribution of CA IV was found in the epididymis, where only epithelial cells of the corpus epididymidis showed CA IV immunoreactivity [21]. A post-testicular transfer of other proteins was demonstrated for sperm adhesion molecule1 (Spam1), which is secreted in epididymosomes released by the epithelial cells to the luminal fluid and integrated into the sperm surface [22], [23]. Such a possible transfer of CA IV is supported by two other findings of the present study.

#### Western-blot and real-time PCR show CI IV mainly in the corpus epididymidis

First western blot analysis shows CA IV only in corpus, cauda and vas deferens and secondly real time-PCR detects mRNA for CA IV only in corpus epididymidis. The amount of transcripts of CA IV mRNA in corpus epididymidis is comparable to that of the kidney, which served as reference. In relation to the mouse kidney, the CA IV mRNA in the corpus epididymidis averages at 54.30% compared to kidney.

The inflow of bicarbonate into the cell can either occur by anion transporters across the cell membrane, or via diffusion of CO_2_, which is then hydrated by intracellular CA. We were able to show CO_2_-induced acceleration of sperm beat frequency in a dose-dependent manner and that treatment with carbonic anhydrase inhibitors EZA or AZA slows acceleration of beat frequency, which shows that CAs are involved in the control of flagellar beating presumably due to hydration of CO_2_ to supply sperm with HCO_3_^−^. The inhibitory effect of AZA in the luminal fluid of epididiymis in rat has been investigated in other studies. It was found that AZA depresses the luminal acidification by 80% in rat cauda epididymidis [24].

#### Murine sperm show significant CA IV activity

In our study, we were primarily interested in characterizing the physiological role of CA IV in murine epididymal spermatozoa. By measuring the response as acceleration of beat frequency of single cells to CO_2_, we show that carbonic anhydrases are involved in bicarbonate supply. The speed of response to CO_2_ could be reduced either by using carbonic anhydrase inhibitors or using sperm of mice with a targeted mutagenesis of the CA IV gene. In spite of other carbonic anhydrases, which are mainly located intracellularly, a state of equilibrium between HCO_3_^−^ and CO_2_ in the cells of CA IV^−/−^ animals takes longer to develop than in sperm of wild-type animals. In addition, the free diffusion of CO_2_ through the sperm membrane appears to be faster than the import of HCO_3_^−^ by anion transporters, which might create a local disequilibrium near the cell surface. When sperm lack CA IV, the reestablishment of the uncatalyzed equilibrium is slower, and acceleration of flagellar beat is delayed. This delay is even more evident when 2% CO_2_ is used for stimulation instead of 5% CO_2_. The presence of other CAs presumably explains why CA activity in sperm of CA IV^−/−^ animals measured by mass spectrometry is reduced only by one third, whereas inhibition of CAs using 5 µM EZA leads to a decrease in enzymatic activity of 62.12%. We discuss this difference either as being an inhibitory effect of EZA towards other CA-isoforms [25], or as a compensatory effect by upregulation of other CA-isoforms during spermatogenesis. It is even more notable that the lack of CA IV is compensated only partially and underlines the importance of CA IV for spermatozoa. It was not surprising that we did not observe fundamental changes in the response to bicarbonate in CA IV^−/−^ animals, since the transport of bicarbonate through anion transporters is not affected in CA IV^−/−^ sperm.

### cAMP acts downstream of CA

The application of cAMP analog cBIMPS in wild-type and CAIV^−/−^ animals increases flagellar beat frequency to a similar extent. As expected, treatment of wild-type sperm with EZA, did not lead to a difference in cBIMPS-induced acceleration of sperm beat, demonstrating that cBIMPS bypasses the action of HCO_3_^−^ on soluble adenylyl cyclase.

### CO_2_ and HCO_3_^−^ show similar effects on human sperm beat frequency

It was previously shown that also human sperm speed flagellar beat in response to HCO_3_^−^ from 6.60 Hz to 12.20 Hz [26]. We extended our studies and successfully demonstrate for the first time that human spermatozoa also respond to CO_2_ with an approximately doubled increase of beat frequency from 7.51 Hz to 14.62 Hz. This response can be reduced with EZA about 27.56% and elucidates carbonic anhydrases to be active in these cells. Beeing responsible for one third of the whole CA activity, CA IV is comparable to membrane bound CA in other cells like astorcytes [27]. However, the fact that CA IV is transferred in the corpus epididymids to the sperm suface accentuates the importance for post-testicular regulation of CO_2_ and HCO_3_^−^ and therefore motility.

The use of computer-assisted sperm analysis (CASA) demonstrates that basic motility parameters of a whole sperm population can be significantly different compared to the evaluation of motility parameters on a single cell level. We found significant differences in the amount of motile and fast progressive spermatozoa between wild-type and CA IV^−/−^ sperm. Determination of the basal beat frequency of motile sperm did not reveal any alterations in CA^−/−^ animals.

### Model of CA IV action in murine sperm

**Figure 8:**
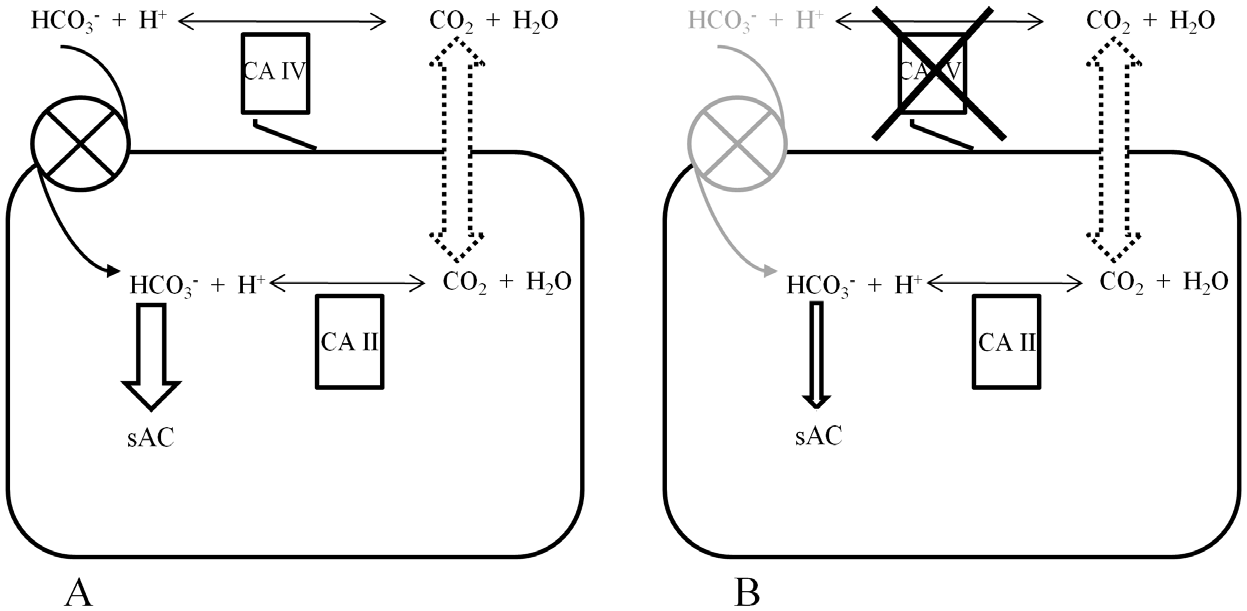
Model of CA IV action in spermatozoa. The extracellular carbonic anhydrase IV (CA IV) equilibrates carbondioxide and bicarbonate close to the sperm membrane. CO_2_ is freely diffusible through the plasma membrane and can be catalyzed by internal CA, such as CA II, to produce bicarbonate. Bicarbonate can also be transported by anion exchangers from the extracellular to the intracellular space (A). Transgenic animals lacking CA IV will experience a delayed equilibrium of CO_2_ and HCO_3_^−^, which might lead to decreased acceleration of flagellar beat in response to external CO_2_ (B).

In conclusion we would postulate the role of CA IV in spermatozoa as follows: CA IV, as an external carbonic anhydrase, equilibrates HCO_3_^−^ and CO_2_ near the sperm surface, so that an increase in external CO_2_ rapidly replenishes HCO_3_^−^ at the extracellular membrane face. Bicarbonate can enter the cell by anion transporters. In sperm carbonic anhydrases located in the cytoplasm, such as CA II, use CO_2_ to provide HCO_3_^−^ by catalytic fast equilibration, resulting in early activation of spermatozoa by sAC. A targeted disruption of CA IV inhibits fast equilibration between HCO_3_^−^ and CO_2_ near the cell membrane and leads to a transient disequilibrium, and delayed restoration of the HCO_3_^−^ concentration resulting in decreased HCO_3_^−^ influx and hence smaller activation of HCO_3_^−^-dependent sAC-mediated activation of flagellar beat (Fig. 8).
